# Isolation, Genomic Characterization and Evolution of Six Porcine Rotavirus A Strains in a Pig Farming Group

**DOI:** 10.3390/vetsci11090436

**Published:** 2024-09-14

**Authors:** Zhendong Zhang, Chengyue Wu, Yue Chen, Yubo Li, Duo Li, Wenqiang Wang, Wei Wen, Zhenbang Zhu, Xiangdong Li

**Affiliations:** 1 Jiangsu Co–Innovation Center for Prevention and Control of Important Animal Infectious Diseases and Zoonoses, College of Veterinary Medicine, Yangzhou University, Yangzhou 225009, China; 008686@yzu.edu.cn (Z.Z.);; 2 School of Biotechnology, Jiangsu University of Science and Technology, Zhenjiang 212100, China; 3 College of Veterinary Medicine, Nanjing Agricultural University, Nanjing 210095, China

**Keywords:** porcine group A rotavirus, genetic diversity, reassortment, recombination, evolution, transmission

## Abstract

**Simple Summary:**

Porcine rotavirus re-emerged in recent years in China. In this study, we analyzed the genomic characterizations of six porcine rotavirus. A strains (PoRVA) isolated from three sow farms in a company at different sampling periods, and in detail dissected their evolutionary relationship. Our results showed transmission and recombination happened frequently within and between pig farms under the same production system. More importantly, several gene segments of isolates came from human rotavirus strains, suggesting the potential cross–species infection between humans and pigs. Our findings provide insights into the transmission and evolution of porcine rotavirus (PoRV) between farms and enrich the molecular epidemiological data of porcine rotavirus in China.

**Abstract:**

Porcine rotavirus (PoRV) is a significant enteric pathogen causing gastroenteritis in piglets, which causes huge economic loss to the Chinese pig industry. In this study, six porcine rotavirus A strains were isolated from three adjacent sow farms belonging to the same company within one year, which suffered severe diarrhea outbreaks. AHBZ2303 (G11P[7]) and AHBZ2305 (G9P[23]), AHBZ2304 (G9P[23]) and AHBZ2312 (G4P[6]), AHBZ2310 (G9P[23]) and AHBZ2402 (G5P[23]) were isolated from Farm A, B and C, respectively. All six isolates were related to human rotavirus through complete genome analysis, suggesting the potential cross–species infection between humans and pigs. Evolutionary analysis revealed that AHBZ2303 and AHBZ2304 likely emerged simultaneously in Farm A and B, and then AHBZ2304 was introduced to Farm A and C, leading to the emergence of AHBZ2305 and AHBZ2310. Recombination and large variation were identified for AHBZ2312 and AHBZ2402. These findings provided insights into the transmission and evolution of PoRV among farms and underscored the need for enhanced monitoring to mitigate the risk of outbreaks from novel variants.

## 1. Introduction

Rotavirus, a non-enveloped and segmented double strand RNA (dsRNA)virus belonging to the family *Sedoreoviridae* (ICTV), stands as a major etiological agent of viral diarrhea in children, young mammals, and birds, resulting in numerous annual child fatalities and adverse economic effects on animal production [1,2,3]. Despite reports indicating a less direct association between rotavirus infection and diarrhea in pigs, these viruses have been posing a persistent challenge to pig health management due to their ubiquitous nature and high resistance in the environment [4,5]. Rotavirus diarrhea outbreaks often afflict intensive sow farms with suboptimal management practices and irregular production, resulting in the mortality of affected piglets or reduced weight gain in surviving pigs [6]. Furthermore, pigs harboring rotavirus have been considered to be crucial interspecies hosts in human infections, driving rotavirus evolution and diversity [7,8]. Among the diverse group of porcine rotavirus species, group A rotavirus (RVAs) remains the most prevalent and widespread viral agent globally [9]. Porcine RVA (PoRVA) was initially identified in 1975, with previous studies reporting PoRVA prevalence ranging from 3.3% to 67.3% in pigs and 61% to 74% at the farm level [1]. In China, surveillance of PoRVAs was limited. Previous studies showed that the prevalence rate of PoRVA was 28.76% in Shandong province [10]. An analysis of molecular characterization data for PoRVAs in East China indicated a 16.8% positivity out of 594 samples collected from both healthy and sick pigs from September 2017 to December 2019 [11]. Based on our surveillance and the latest reports performed by Qiao et al. [12], the prevalence of PoRVs has been increasing in Chinese pig herds in recent years, posing a potentially more severe threat to the local pig industry.

Before 1984, isolation of RVs in the cell culture was quite challenging until African Green Monkey kidney cells (MA104) were utilized to cultivate the virus in the presence of trypsin [13]. Eleven gene fragments encode 6 structural proteins (VP1–VP4, VP6, and VP7), and 5 or 6 nonstructural proteins (NSP1–NSP5 or NSP6) from the genome of rotavirus. As more human and animal rotavirus genomes were sequenced, and to better define the genotypes of rotavirus, the Rotavirus Classification Working Group (RCWG) proposed the classification system of RVs based on the nucleotide identity cut–off percentages, in which Gx–P[x]–Ix–Rx–Cx–Mx–Ax–Nx–Tx–Ex–Hx are used for the VP7–VP4–VP6–VP1–VP2–VP3–NSP1–NSP2–NSP3–NSP4–NSP5/6 encoding genes, respectively [14,15]. Among these classification systems, the dual (G/P) typing system is widely employed to differentiate strains for the change of neutralizing antibody responses induced by the outer shell proteins VP7 and VP4 [16]. Twelve G genotypes and sixteen P genotypes of PoRVA have been associated with swine infections or diseases [1]. PoRVA strains with G3, G4, G5, G9, and G11 genotypes in combination with P[5], P[6], P[7], P[13], P[28], and P[32] are found common in pigs [17]. It is very difficult to eradicate RVAs because of their nature of resistance to disinfection. Good management practices coupled with vaccination might be the priority for controlling the disease [18]. However, continuously emerging mutant and reassortant PoRVAs, along with the high degree of genetic and antigenic variation, exacerbate clinical complexity and weaken the efficacy of commercial vaccines [19,20]. Hence, strengthening surveillance and understanding the evolution of PoRVAs can facilitate the development of more potent vaccines to control the disease and alleviate the global burden of RVAs.

In the present study, we collected fecal samples from visibly diarrheic piglets at three sow farms and continuously tracked the virus dynamics and the evolution of dominant rotavirus. Interestingly, two different G- and P- genotype RVA strains were isolated from MA104 cells at each of the three farms, and the evolution and genetic variation of six isolates were analyzed based on their genomic sequences. The results provide valuable insights into RVA’s evolution at the farm level, enhance our understanding of epidemic characteristics data, and facilitate future investigations and control measures for PoRVAs.

## 2. Materials and Methods

### 2.1. The Background Information of Diarrheic Samples and Pathogens Detection

Three intensive sow farms in Anhui Province of China experienced outbreaks of clinical diarrhea among suckling piglets with morbidity rates ranging from 20% to 60%. The clinical diarrhea lasted for 3–5 days and no deaths of infected piglets were reported. The yellow watery or semisolid feces were collected from these three farms on different dates (farm A in March and May 2023, farm B in April and December 2023, and farm C in October 2023 and February 2024). The diarrheic samples were reconstituted with phosphate buffer saline (PBS) at a ratio of 1:5, followed by clarification through centrifugation at 8000× *g* at 4 °C for 10 min. The supernatant was subjected to nucleic acid extraction using the Simply P Viral DNA/RNA Kit (Bioer Technology, Hangzhou, China) according to the manufacturer’s instructions. The commercial RT-qPCR kit (Jiazhi, Qingdao, China) was performed to detect the common pathogens associated with diarrhea for piglets, including porcine epidemic diarrhea virus (PEDV), porcine transmissible gastroenteritis virus (TGEV), PoRVA, PoRVB and PoRVC, and porcine deltacoronavirus (PDCoV). The positive samples for rotavirus were then amplified using the RT–PCR method based on the designed VP6 and VP7 primers (Supplementary Table S1). Further, porcine reproductive and respiratory syndrome virus (PRRSV) and porcine circovirus 2 (PCV2) were also detected using the qPCR method established in our lab.

### 2.2. Cells, Virus Isolation and Immunofluorescence Assay (IFA)

MA104 cells cultured in Medium 199 (M199) (Gibco, Shanghai, China) supplemented with 10% fetal bovine serum (NULEN BIOTECH, Shanghai, China) at 37 °C in a humidified incubator under 5% CO_2_ were used for rotavirus isolation. The supernatant of the clinical positive rotavirus samples (ct < 18) was decontaminated using 0.22 μm filter, then mixed with M199 containing 20 μg/mL trypsin (Sigma, St. Louis, MO, USA) at a 1:5 ratio and incubated at 37 °C for one hour, which was isolated through passaging in MA104 cell monolayers in the presence of trypsin (10 μg/mL) modified as previously described [21]. The third passage of each isolate was purified using the limited–dilution method twice, which then were identified by RT–PCR and indirect immunofluorescence assay (IFA) with the polyclonal antibody specific for VP6 protein prepared in our lab. The infected MA104 cells cultured in 6-well plates were subjected to IFA assay as the protocol that was published before [21,22].

### 2.3. RT–PCR Amplification, Genome Sequencing, and Genotyping

MA104 cells were inoculated with the purified virus isolates. Viral RNA was extracted from the virus supernatant using TRIzol reagent (Invitrogen, Carlsbad, CA, USA). The RNA was reverse transcribed into cDNA using HisScript II 1st Strand cDNA Synthesis Kit (Vazyme, Nanjing, China) to amplify the full genome of eleven segments of rotavirus by using 2 × Taq Master Mix (Vazyme, Nanjing, China). The 5′ and 3′ regions of each segment were obtained using 5′ RACE kits (Takara, Dalian, China) as described before [23]. Based on the acquired 5′ and 3′ sequences, the primers used in the study were designed using PoRVA genomic sequences available in GenBank and presented in Supplementary Table S1. The genotypic constellations of the isolated strains were performed and determined using the online ViPR-tool (https://www.rivm.nl/mpf/typingtool/rotavirusa/, accessed on 13 December 2023) along with BLASTn analysis (NCBI).

### 2.4. Homology Alignments and Phylogenetic Analysis of Each Segment

To understand the evolutionary characteristics of the isolates, nucleotide and deduced amino acid alignments were constructed using the multiple sequence alignment program (Clustal W), and eleven phylogenetic trees were constructed based on the complete genomic segments of the eleven rotaviruses, together with several related representative strains (Supplementary Table S2), respectively. Phylogenetic trees and molecular evolutionary analyses were carried out by the MEGA 11 software using the maximum likelihood (ML) method with the 1000 bootstrapped replicates.

### 2.5. Recombination Analysis of Six Isolates

To analyze possible recombination events, every single segment of the six isolates was selected for RDP4 software version 4 and SimPlot analysis. The recombinant segments confirmed with two software were displayed based on similarity plot analysis implemented in SimPlot v3.5.1 within a 200-bp window sliding along the sequence alignment (20-bp step size) as described previously [22,24].

## 3. Results

### 3.1. Detection, Isolation, and Identification of PoRVA

**Figure 1:**
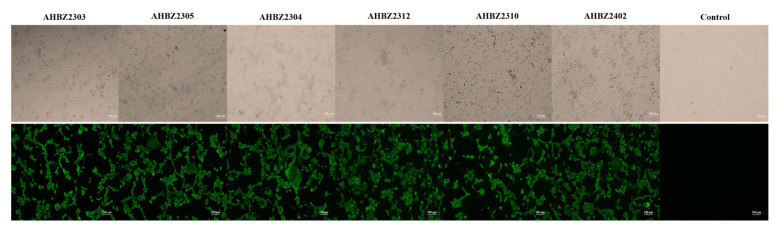
Isolation and identification of six porcine rotavirus A strains. Cytopathic effects (CPE) infected with the six isolates and immunostaining (green) analysis of VP6 protein in MA104 cells.

Out of 67 diarrheic samples obtained from the three sow farms, 53 (79.1%) were positive for PoRVA via qPCR and RT–PCR detections with no presence of any other common pathogens including PEDV, TGEV, PDCoV, RVB, RVC, PRRSV and PCV2. Twelve positive samples of RVA (CT < 18) from different times and farms were inoculated into MA104 cells for three blind passages to isolate the virus. Six strains were successfully isolated and showed obvious cytopathic effects (CPEs) on MA104 cells characterized by cell rounding, elongation, and detachment by the first or second generations (Figure 1). Furthermore, the isolated strain was confirmed by the detection of PoRVA antigens with IFA using PoRVA–VP6–specific polyclonal antibodies. At 48 h post–infection (hpi), specific green signals were observed in the infected MA104 cells, but no fluorescence was detected in the control (Figure 1). The isolates were purified using the limited–dilution method twice at passage 3 and were designated as AHBZ2303, AHBZ2304, AHBZ2305, AHBZ2310, AHBZ2312, and AHBZ2402 based on the isolate location and time (2303 signifies March 2023 and 2310 indicates October 2023, and so forth). The strains AHBZ2303 and AHBZ2305, AHBZ2304 and AHBZ2312, and AHBZ2310 and AHBZ2402 were isolated from farms A, B, and C at different sampling periods, respectively (Table 1). 

### 3.2. Whole Genome Sequencing and Genotyping

The complete sequences for all segments of six isolated PoRVA strains were determined using the primers as shown in Supplementary Table S1, and the sequences of VP6, VP7 and VP4 were deposited in GenBank with the accession numbers PP683066–PP683068 for AHBZ2303, PP683072–PP683074 for AHBZ2305, PP683069–PP683071 for AHBZ2304, PP682346–PP682348 for AHBZ2312, PP683075–PP683077 for AHBZ2310, PP683078–PP683080 for AHBZ2402, respectively. According to the classification of RCWG, 80% (G), 80% (P), 85% (I), 83% (R), 84% (C), 81% (M), 79% (A), 85% (N), 85% (T), 85% (E), and 91% (H) nucleotide identity cutoff values were used to differentiate 11 gene segments of RVAs. Analysis of the complete genome sequence using the online ViPR–tool revealed that four G–genotypes (G9, G4, G5, and G11), three P–genotypes (P[23], P[7], and P[6]), two I–genotypes (I5 and I1) and the same constellation (R1–C1–M1–A8–N1–T1–E1–H1) of the other eight genes were identified for the six isolates (Table 1), which was similar with the results reported by Qiao et al. [12]. Interestingly, different combinations of VP7 and VP4 were observed in all three farms at different times, and the I–genotypes changed from I5 to I1 in Farm B after 8 months, implying that the dominant virus in the three farms has evolved and undergone large variation.

### 3.3. Homology Alignments and Phylogenetic Analyses

BLASTn analyses of the individual segments were performed and revealed that all six isolates show obvious reassortment events on the backbone of porcine rotavirus with one, two, or three segments derived from human rotavirus (Table 2). The segment 1–encoding VP1 of AHBZ2303 and AHBZ2310 exhibited highest identities with human rotavirus E931 strain, the segment 3–encoding VP3 of AHBZ2303 and AHBZ2402 had an even higher homology with human rotavirus strains, and segment 10–encoding nsp4 of AHBZ2303 was most closely related to the human rotavirus strain R479. The nsp2 and nsp5 genes of four isolates shared more than 98% nucleotide identity with human rotavirus, suggesting that this reassortment event might be beneficial for rotavirus replication [25,26]. Likewise, porcine–porcine reassortment events were also identified for the six isolates, as the other segments from porcine showed different similarities with different strains. 

The nucleotide and amino acid identities of isolated rotavirus from the same farm were analyzed (Table 3). In Farm A, the VP7, VP4, and VP3 genes of AHBZ2305 showed 75.4%, 73.9%, and 84.8% nucleotide sequence identity with previous AHBZ2303, with less difference for the other eight segments–coding genes. In Farm B, the VP7, VP4, VP6, VP2, and NSP2 genes of AHBZ2312 exhibited more than a 10% difference with AHBZ2304, and only 66.3% nucleotide homology was identified for the VP6 gene, which could be introduced from other rotavirus from outside or due to the longer evolution time for the AHBZ2312 (more than 8 months). All the eleven segments–encoding genes of AHBZ2402 were not very close to the strain AHBZ2310 isolated from the same farm C, with the highest nucleotide sequence identity being 97.1% in NSP5, implying new introduction or large mutation or recombination occurred after AHBZ2310. Farms A, B, and C belonged to the same company and were located in neighboring areas, so the relationship of the isolates among farms was also analyzed. For VP7, VP4, VP6, VP1, VP2, VP3, NSP1, NSP2, NSP3, NSP4, and NSP5, the six isolates showed 73.6–99.8%, 66.3–99.8%, 82.2–99.3%, 86.2–100%, 86.7–100%, 84.8–99.8%, 81.3–99.9%, 89.5–100%, 95.8–100%, 89.2–100%, and 96.8–100% nucleotide identities, respectively (Supplementary Tables S3–S13). Except for VP7, VP4, VP6, and VP3, the other seven genes of AHBZ2303 isolated from Farm A shared high identities (98.1% for NSP4, 99.9–100% for others) with AHBZ2304 isolated from Farm B, so we speculated that these two isolates maybe derive from one same parental virus and undergo different evolution or reassortment events.

**Figure 2:**
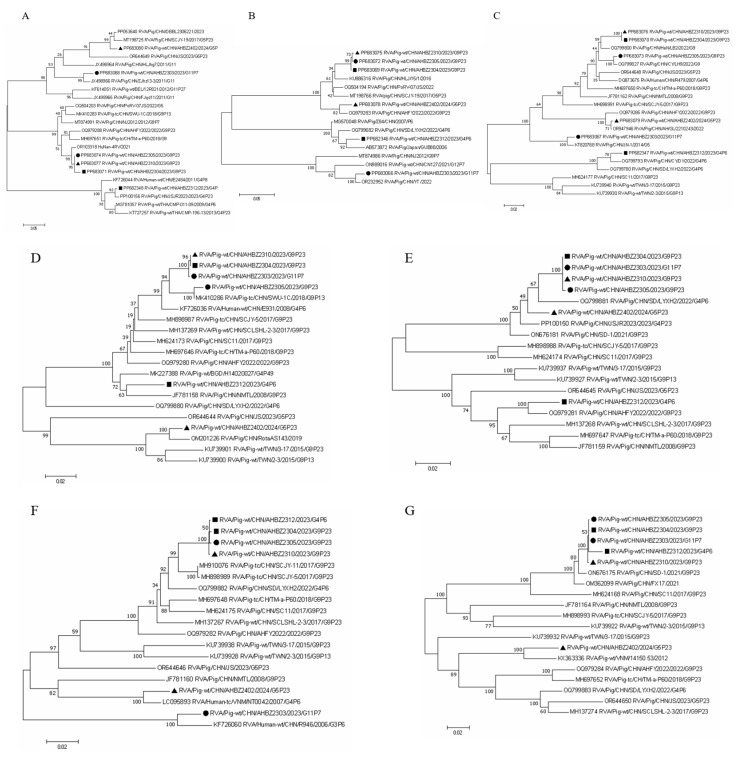

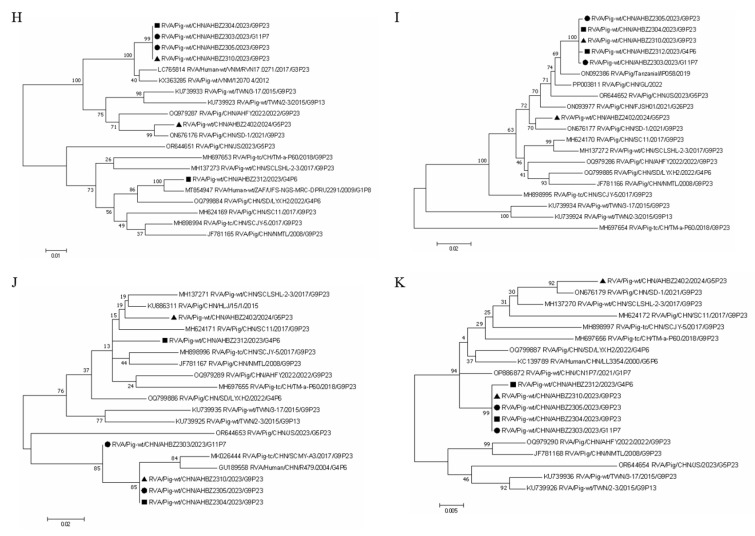
Phylogenic trees of the six isolates and other representative strains. The phylogenic trees of (**A**–**K**) were constructed based on the VP7, VP4, VP6, VP1, VP2, VP3, NSP1, NSP2, NSP3, NSP4, and NSP5 genes by MEGA X using the neighbor–joining (NJ) method with 1000 bootstrap replicates, respectively. The strains isolated from Farm A are labeled with black box. The strains isolated from Farm B are labeled with black circles. The strains isolated from Farm C are labeled with black triangles.

To have a better knowledge of the evolution of the six isolates, phylogenetic analyses based on the nucleotide sequences were conducted using the closest and several related reference strains. As shown in Figure 2, all eleven segments of the six isolates were clustered into the same clade with the corresponding reference strains (Figure 2), which demonstrated the prevailing reassortment events for porcine rotavirus, similar to BLAST results (Table 2). For VP7, VP4, and VP6, four separate branches were formed based on the six isolates from different farms, and interestingly, AHBZ2304, AHBZ2310, and AHBZ2305 isolated from different farms were closer to each other and clustered into the same branch, providing the evidence of cross–transmission among different farms. Furthermore, most of the segments of AHBZ2312 and AHBZ2402 isolated from Farm B and C were farther away from the other four isolates and clustered into separate branches, which implied that the extended evolution or other unexpected events occurred in the same farm.

### 3.4. Reassortment and Recombination Analysis among the Six Isolates

**Figure 3:**
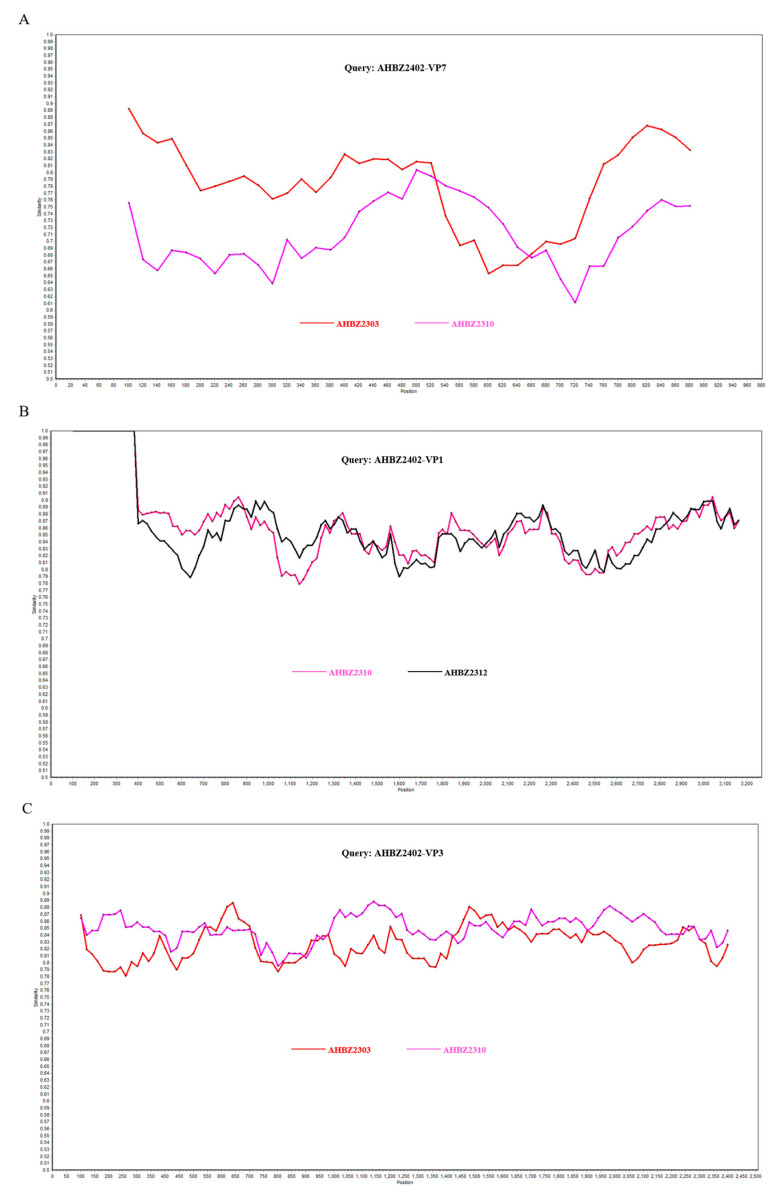
Recombination analysis of VP7 (**A**), VP1 (**B**), and VP3 (**C**) gene of AHBZ2402. Comparisons of genetic similarity between recombinant and potential parental segments were made using SimPlot.

AHBZ2305 was isolated from Farm A in May 2023, two months later than AHBZ2303. However, based on the complete genome of all eleven segments, AHBZ2305 was most closely related to AHBZ2304 (96–100% nucleotide identities), except for several genes showing high identities with AHBZ2303 (Table 4). So, combing the results of phylogenetic trees (Figure 2), we speculated that AHBZ2305 was very likely derived from AHBZ2304 directly, or underwent reassortment events between these two viruses (Table 4). Coincidentally, phylogenetic analysis showed that all the segments of AHBZ2304, AHBZ2310, and AHBZ2305 clustered in a large branch (Figure 2), and ten segment genes of AHBZ2310 exhibited high identities with AHBZ2304 (99.3–100%) and one with AHBZ2305 (Table 4). Strong evidence demonstrated that AHBZ2310 isolated from Farm C most likely derived from AHBZ2304 directly, or acquired ten segments from AHBZ2304 and one segment (VP7) from AHBZ2305. To analyze the potential recombination events among the six isolates, each segment of the isolates was queried using RDP4 and SimPlot software. As shown in Figure 3, three recombinant segments were identified. The VP7 and VP3 of AHBZ2402 were recombinant segments that emerged from the recombination event between AHBZ2303 and AHBZ2310, and the VP1 of AHBZ2402 was a recombinant derived from AHBZ2310 and AHBZ2312. 

### 3.5. Neutralizing Epitope Analysis of VP7 and VP4

**Figure 4:**
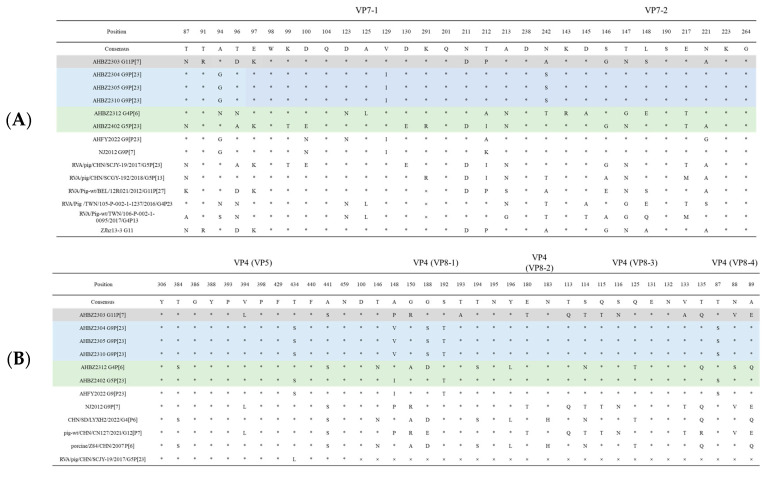
Neutralizing epitopes on the VP7 (**A**) and VP4 (**B**) proteins in different strains. The different letters mean the amino acids and the “×” represents the deletion of the amino acids, “*” represent the same amino acids.

Neutralizing epitopes of VP7 and VP4 protein from the six isolates were compared with each other and several referenced strains as described before [23,27]. As shown in Figure 4, among the six isolates, seven amino acids (98W, 104Q, 201Q, 238D, 190S, 223K, and 264G) were found to be conserved within the neutralizing epitopes in VP7. In VP5 and VP8 cleaved from VP4 protein [28], eight amino acids (306Y, 386G, 388Y, 393P, 398P, 429F, 440F, and 459N) and five amino acids (100D, 195N, 183N, 131E, and 132N) showed conservation, respectively. Less conservation of the main neutralizing epitopes demonstrated the potential characteristics of rapid mutation of rotavirus. For AHBZ2304, AHBZ2310, and AHBZ2305, all the amino acids of neutralizing epitopes were identical to each other, further suggesting that AHBZ2310 and AHBZ2305 maybe derived from AHBZ2304 in the same production system. At the farm level, for AHBZ2303, AHBZ2312, and AHBZ2402, a larger difference was identified compared to the porcine rotavirus (AHBZ2305, AHBZ2304, and AHBZ2310, respectively) isolated from the same farm, so we speculated that the escape of neutralizing antibody may result to the emergence of new strains. Compared to the other four isolates, AHBZ2303 and AHBZ2402 possessed the specific seven amino acids (87N, 94A, 97K, 211D, 146G, 147N, and 221A) within the neutralizing epitopes in VP7, indicating the potential relationship between these two viruses. Moreover, AHBZ2312 exhibited three new amino acid variations (291K, 212A, and 143R) compared to two referenced G4 rotavirus, showing mutation continues to occur in the field. 

## 4. Discussion

PoRV infection leading to clinical diarrhea has been increasing in recent years, which poses new challenges to the swine industry in China. Our analysis of domestic and foreign literature related to Chinese PoRV revealed that 37 PoRVA strains have been isolated from 33 studies, with more than two–thirds of the isolates being acquired after 2018, which strongly indicates the increasing epidemic situation warranting increased attention from researchers and producers. Qiao et al. performed a nationwide epidemiological investigation of PoRVA in 2022, and the results showed that 86.52% of the pig farms tested positive, with an overall positive rate of 51.15% in China [12]. Further genetic evolution analysis revealed that G9, P[13], and I5 were the predominant genotype and G9P[23] was determined as the most prevailing genotype combination [12], which was consistent with the results based on the 37 PoRVA strains. In this study, six PoRVA strains were successfully isolated from three neighboring sow farms experiencing diarrhea under the same company. Four G–genotypes (G9, G4, G5, and G11), three P–genotypes (P[23], P[7], and P[6]), and two I–genotypes (I5 and I1) were determined through the genetic analysis of VP7, VP4, and VP6 genes, highlighting the diversity and complexity of the predominant virus in these farms. 

Reassortment and recombination play crucial roles in rotavirus evolution, particularly between pigs and humans. Yan et al. isolated three PoRVAs in one sow farm and ten gene segments of the pig–breeder isolate were closely related to the pig isolate, providing evidence of interspecies transmission of PoRV from pigs to humans [29]. Wu et al. surveyed 4588 porcine stool samples from 2014 to 2017 in Taiwan and demonstrated that pigs serve as a reservoir for potential zoonotic transmission [8]. Analyzing Chinese 36 PoRVA isolates since 2008, we found that only one strain AY01 showed no reassortment events, and one or more genes of more than 90% of isolates exhibited the highest identities with human rotavirus (data not shown). Here, all six isolates showed high homology to human RVA strains across VP1, VP3, NSP2, NSP4, and NSP5 (Table 2). Notably, in four of the six isolates, the nsp2 and nsp5 genes shared more than 98% nucleotide identity with human rotavirus. The underlying mechanism warrants further study. Additionally, the homology alignments and phylogenetic analyses revealed the reassortment events of six isolates also occurred from different PoRVA strains. Based on the complete genome analysis, we speculated that AHBZ2305 and AHBZ2310 underwent potential reassortment between AHBZ2303 and AHBZ2304, as well as between AHBZ2304 and AHBZ2305 (Table 2). Parra et al. first reported evidence of intragenic recombination [30], and Hoxie et al. analyzed 23,627 complete rotavirus genome sequences available in the NCBI and found strong evidence for recombination in nine of eleven rotavirus A segments (except NSP3 and NSP5) [31]. In our study, three obvious recombination events were determined for AHBZ2402 (Figure 3). More interesting, the VP7 gene of AHBZ2402 (G5 genotype) was produced from AHBZ2303 (G11 genotype) and AHBZ2310 (G9 genotype), providing evidence of recombination event between two strains of the different rotavirus genotype. The above results enriched the knowledge about porcine rotavirus evolution and divergence and more future investigations should be performed for better understanding the transmissibility between humans and pigs.

Segment 9 and 4 encoding VP7 and VP4 serve as the main neutralizing antigen against rotavirus infection and are commonly targeted in molecular epidemiological investigations. In the dual typing system, different G and P genotypes were identified based on the 80% nucleotide identity threshold, suggesting significant differences may exist even within the same G or P genotype. Lineage analysis is typically performed to study genetic evolution across different viruses [32], so we suggested more refined classifications are needed in the future for porcine rotavirus. Due to the limited classification details, we only selected several referenced strains to perform the phylogenetic tree and focused primarily on the evolution analysis among the six isolates. Furthermore, given that these three farms belonged to the same company and were situated in neighboring areas, we paid particular attention to understanding the relationship among isolates from different farms (Supplementary Figure S1). Based on outbreak time and genetic analysis, we strongly suspect that Farm A and B were infected around the same time, with the isolation of AHBZ2303 and AHBZ2304. Then, Farm A and C likely introduced the porcine rotavirus AHBZ2304, as evidenced by the high homology shared between AHBZ2304, AHBZ2305 (Farm A), and AHBZ2310 (Farm C), with all the eleven genes of clustering into the same branch (Figure 2). SimPlot analysis showed that AHBZ2402 isolated from Farm C may be produced from the recombination events between the isolates from the other two farms. Our results provided the direct epidemiological link demonstrating the transmission of porcine rotavirus among farms. According to farm veterinarians, although there was only transient infection without fatalities occurring among the sucking piglets, and no obvious differences in morbidity rates or clinical syndromes were observed among different isolates, the drying of barns, timely care of diarrheal piglets, and other management measures were very important for the control of the rotavirus–related diseases in the field. Further exploration of their pathogenicity and clinical aspects is necessary for better elucidating the impact of rotavirus on pig production.

## 5. Conclusions

Taken together, six porcine rotavirus A strains were successfully isolated from three adjacent sow farms. Genomic and evolutionary analyses reveal the characteristics of porcine rotavirus with broad variation and rapid evolution and provide direct evidence of potential inter–farm transmission. Our findings enrich the molecular epidemiological data of porcine rotavirus in China and emphasize the significant importance of PoRV monitoring and control in the field.

## Figures and Tables

**Table 1:** Genotype constellation of the six isolated porcine group A rotaviruses.

Farm	Sampling Time	Isolates	Genotypes										
			VP7	VP4	VP6	VP1	VP2	VP3	NSP1	NSP2	NSP3	NSP4	NSP5
A	March 2023	AHBZ2303	G11	P[7]	I5	R1	C1	M1	A8	N1	T1	E1	H1
	May 2023	AHBZ2305	G9	P[23]	I5	R1	C1	M1	A8	N1	T1	E1	H1
B	April 2023	AHBZ2304	G9	P[23]	I5	R1	C1	M1	A8	N1	T1	E1	H1
	December 2023	AHBZ2312	G4	P[6]	I1	R1	C1	M1	A8	N1	T1	E1	H1
C	October 2023	AHBZ2310	G9	P[23]	I5	R1	C1	M1	A8	N1	T1	E1	H1
	February 2024	AHBZ2402	G5	P[23]	I5	R1	C1	M1	A8	N1	T1	E1	H1

**Table 2:** Closest sequences to the six isolates from GenBank database.

Gene	Closest Strain (Host, Name, Identity)					
	Farm A		Farm B		Farm C	
	AHBZ2303	AHBZ2305	AHBZ2304	AHBZ2312	AHBZ2310	AHBZ2402
VP7	Porcine ZJhz13–3 94.47%	Porcine HuNan–4RV 99.08%	Porcine HuNan–4RV 98.06%	Porcine HuNan–4RV 97.86%	Porcine HuNan–4RV 97.86%	Porcine DB/BL/2306221 98.06%
VP4	Porcine YT 97.89%	Porcine HLJ/15/1 95.59%	Porcine HLJ/15/1 95.59%	Porcine GUB88 95.81%	Porcine HLJ/15/1 95.80%	Porcine AHFY2022 97.34%
VP6	Porcine JN–1 98.43%	Porcine LH9 99.25%	Porcine LH9 99.25%	Porcine DY 97.91%	Porcine LB2 96.57%	Porcine GL 99.75%
VP1	**Human E931 97.35%**	Porcine SWU–1C 97.12%	Porcine SWU–1C 99.06%	Porcine H14020027 95.62%	**Human** **E931** **97.22%**	Porcine S143 97.92%
VP2	Porcine SD–1 96.07%	Porcine SD–1 95.92%	Porcine SD–1 95.92%	Porcine AHFY2022 97.04%	Porcine SD–1 96.26%	Porcine JSJR2023 97.42%
VP3	**Human R946 96.45%**	Porcine SCJY–11 96.61%	Porcine SCJY–11 96.61%	Porcine SCJY–11 96.97%	Porcine SCJY–11 96.97%	**Human** **NT0042** **97.33%**
NSP1	Porcine FX17 97.72%	Porcine SD–1 98.15%	Porcine SD–1 98.15%	Porcine SD–1 97.34%	Porcine SD–1 98.29%	Porcine 14150_53 94.39%
NSP2	Porcine 12070_4 97.60%	**Human RVN17.0271 98.11%**	**Human RVN17.0271 98.11%**	**Human DPRU1554 98.01%**	**Human RVN17.0271 98.11%**	Porcine SD–1 98.43%
NSP3	Porcine IP058 97.75%	Porcine GL 97.56%	Porcine GL 97.56%	Porcine GL 97.66%	Porcine GL 97.88%	Porcine SD–1 97.32%
NSP4	**Human R479 94.80%**	Porcine SCMY–A3 96.40%	Porcine SCMY–A3 96.40%	Porcine HLJ/15/1 95.64%	Porcine SCMY–A3 96.40%	Porcine HLJ/15/1 96.78%
NSP5	Porcine CN1P7 98.23%	**Human LL3354 98.48%**	**Human LL3354 98.48%**	**Human LL3354 98.15%**	**Human LL3354 98.48%**	Porcine SD–1 98.82%

Closest sequences from human rotavirus are indicated in bold.

**Table 3:** Nucleotide and amino acid identities of genomic segments isolated from same farm.

Farm	Query Strain	Pairwise % Identity (Nucleotide, Amino Acid)										
		VP7	VP4	VP6	VP1	VP2	VP3	NSP1	NSP2	NSP3	NSP4	NSP5
A	AHBZ2303	75.4	73.9	90.5	96.6	99.3	84.8	99.9	99.9	99.6	98.1	100
	AHBZ2305	84.4	81.1	98.0	97.5	98.8	93.4	99.8	100	99.4	98.9	100
B	AHBZ2304	76.6	66.3	82.6	93.3	87.2	99.8	98.6	89.6	99.8	91.1	99.7
	AHBZ2312	80.4	62.4	94.7	99.0	97.5	99.8	96.9	96.2	100	95.5	100
C	AHBZ2310	77.8	92.4	88.1	86.5	96.9	86.5	82.0	93.7	96.1	89.2	97.1
	AHBZ2402	83.5	96.8	96.5	96.8	99.0	94.0	82.1	95.3	99.4	97.2	99.5

**Table 4:** Nucleotide identities of potential reassortant strain AHBZ2305 and AHBZ2310.

Query Strain	Potential Parental Strain	Pairwise % Identity (Nucleotide, Amino Acid)										
		VP7	VP4	VP6	VP1	VP2	VP3	NSP1	NSP2	NSP3	NSP4	NSP5
AHBZ2305	AHBZ2303	75.4	73.9	90.5	96.6	99.3	84.8	99.9	99.9	99.6	98.1	100
	AHBZ2304	**98.3**	**99.8**	**96.0**	96.6	99.3	**99.7**	99.9	99.9	99.7	**100**	100
AHBZ2310	AHBZ2304	98.5	**99.8**	**99.3**	**100**	**99.9**	**99.8**	**99.7**	**100**	**100**	**100**	**100**
	AHBZ2305	**99.8**	99.7	95.6	96.6	99.2	99.7	99.7	99.9	99.7	100	100

## Data Availability

The original contributions presented in the study are included in the article/Supplementary Materials, further inquiries can be directed to the corresponding author.

## Supplementary Materials

The following supporting information can be downloaded at: https://www.mdpi.com/article/10.3390/vetsci11090436/s1, Figure S1: The model of the transmission of the six isolates among the three farms; Table S1: Amplification primers used for PoRVAs complete genome; Table S2: The referenced strains used in the present study; Tables S3–S13: The nucleotide identity of eleven genes between the six isolates.
