# Supplementary material for: Isolation, Genomic Characterization and Evolution of Six Porcine Rotavirus A Strains in a Pig Farming Group
Source: Vet Sci. 2024 Sep 14;11(9):436. doi: 10.3390/vetsci11090436 (PMC11435977; doi:10.3390/vetsci11090436)
Supplement: Supplementary file 1 [file vetsci-11-00436-s001.zip › vetsci-3197019-supplementary/supplementary files/Supplementary Table S1.pdf]

Supplementary Table S1. Amplification primers used for PoRVAs complete genome

Gene	Primer	Primer sequence (5'-3')	Size (bp)
VP1	VP1-F	GGCTATTAAAGCTRTACAATGG	3302
	VP1-R	GGTCACATCTAAGCRYTCTAAT	
VP2	VP2-F	GGCTATTAAAGGYTCAATGGCG	2718
	VP2-R	GGTCATATCTCCACARTGGGGT	
VP3	VP3-F	GGCTWTTAAAGCARTAYYAGTAG	2591
	VP3-R	GGTCAYATCRTGACYAGTGTG	
VP4	VP4-F	ATGGCTTCRCTHATTTAYAG	2300
	VP4-R	TTACARTCTACAYTGYA	
VP6	VP6-F	GGCTTTWAAACGAAGTCTTC	1209
	VP6-R	GGTCACATCCTCTCACT	
VP7	VP7-F	GGCTTTAAAAGAGAGAATTTCCG	1060
	VP7-R	GGTCACATCATACARTTCTAA	
NSP1	NSP1-F	GGCTTTTTTTATGAAAAGTCTTGTG	1566
	NSP1-R	GGTCACATTTTATGCTGCCTAG	
NSP2	NSP2-F	GGCTTTTAAAGCGTCTCAGTC	1059
	NSP2-R	GGTCACATAAGCGCTTTCTATTC	
NSP3	NSP3-F	GGCTTTTAATGCTTTTCAGTGGTTG	1074
	NSP3-R	GGTCACATAACGCCCCTATAG	
NSP4	NSP4-F	TGTTCCGAGAGAGCGCGTG	740
	NSP4-R	GACCATTCCTTCCATTAAC	
NSP5	NSP5-F	GGCTTTTAAAGCGCTACAG	664
	NSP5-R	GGTCACAAAACGGGAGT	
