# Supplementary material for: Isolation, Genomic Characterization and Evolution of Six Porcine Rotavirus A Strains in a Pig Farming Group
Source: Vet Sci. 2024 Sep 14;11(9):436. doi: 10.3390/vetsci11090436 (PMC11435977; doi:10.3390/vetsci11090436)
Supplement: Supplementary file 1 [file vetsci-11-00436-s001.zip › vetsci-3197019-supplementary/supplementary files/Supplementary Table S2.pdf]

Supplementary Table S2. The referenced strains used in the present study

Referenced Strains	Genbank Number										
	VP1	VP2	VP3	VP4	VP6	VP7	NSP1	NSP2	NSP3	NSP4	NSP5
RVA/Pig/CHN/NMTL/2008/G9P23	JF781158	JF781159	JF781160		JF781162		JF781164	JF781165	JF781166	JF781167	JF781168
RVA/Pig-wt/TWN/2-3/2015/G9P13	KU739900	KU739927	KU739928		KU739930		KU739922	KU739923	KU739924	KU739925	KU739926
RVA/Pig-wt/TWN/3-17/2015/G9P23	KU739901	KU739937	KU739938		KU739940		KU739932	KU739933	KU739934	KU739935	KU739936
RVA/Pig-wt/CHN/SCLSHL-2-3/2017/G9P23	MH137269	MH137268	MH137267				MH137274	MH137273	MH137272	MH137271	MH137270
RVA/Pig/CHN/SC11/2017/G9P23	MH624173	MH624174	MH624175		MH624177		MH624168	MH624169	MH624170	MH624171	MH624172
RVA/Pig-tc/CH/TM-a-P60/2018/G9P23	MH697646	MH697647	MH697648		MH697650	MH697651	MH697652	MH697653	MH697654	MH697655	MH697656
RVA/Pig-tc/CHN/SCJY-5/2017/G9P23	MH898987	MH898988	MH898989		MH898991		MH898993	MH898994	MH898995	MH898996	MH898997
RVA/Pig/CHN/AHFY2022/2022/G9P23	OQ979280	OQ979281	OQ979282	OQ979283	OQ979285	OQ979288	OQ979284	OQ979287	OQ979286	OQ979289	OQ979290
RVA/Pig/CHN/JS/2023/G5P23	OR644644	OR644645	OR644646		OR644648	OR644649	OR644650	OR644651	OR644652	OR644653	OR644654
RVA/Pig/CHN/SD/LYXH2/2022/G4P6	OQ799880	OQ799881	OQ799882	OQ799682	OQ799780		OQ799883	OQ799884	OQ799885	OQ799886	OQ799887
RVA/Human-wt/CHN/E931/2008/G4P6	KF726036										

RVA/Pig-wt/BGD/H14020027/G4P49	MK227388										
RVA/Pig-tc/CHN/SWU-1C/2018/G9P13	MK410286					MK410283					
RVA/Pig/CHN/SD-1/2021/G9P23		ON676181					ON676175	ON676176	ON676177		ON676179
RVA/Pig/CHN/JSJR2023/2023/G4P23		PP100150				PP100156					
RVA/Human-wt/CHN/R946/2006/G3P6			KF726060								
RVA/Human-tc/VNM/NT0042/2007/G4P6			LC095893								
RVA/Pig-tc/CHN/SCJY-11/2017/G9P23			MH910076								
RVA/Pig/Japan/GUB88/2006				AB573872							
RVA/Pig/CHN/HLJ151/2016				KU886316						KU886311	
RVA/Pig/CHN/PoRV-07JS/2022				OQ504194		OQ504203					
RVA/Pig/CHN/YT/2022				OR232952							
RVA/Pig/CHN/Z84/2007/P6				MG570048							
RVA/Pig/CHN/SCJY-19/2017/G5P23				MT198766		MT198725					

RVA/Pig/CHN/NJ/2012/G9P7	MT874986		MT874991
RVA/Pig-wt/CHN/CN127/2021/G12P7	ON989016		
RVA/Human/CHN/R479/2007/G4P6		DQ873675	
RVA/Pig/CHN/JN-1/2014/G5		KT820768	
RVA/Pig/CHN/CY/DY/2022/G4P6		OQ799793	
RVA/Pig/CHN/HeN/LB2/2022/G9		OQ799800	
RVA/Pig/CHN/CY/LH9/2022/G9		OQ799827	
RVA/Pig/CHN/AH/GL/2210243/2022		OR947946	
RVA/Pig/CHN/HLJhg7/2011/G11			JX498964
RVA/Pig/CHN/FJqd11/2011/G11			JX498965
RVA/Pig/CHN/ZJhz13-3/2011/G11			JX498966
RVA/Pig-wt/BEL/12R021/2012/G11P27			KF614051
RVA/Human-wt/CHN/E2484/2011/G4P8			KF726044

RVA/Pig-wt/THA/CMP-196-13/2013/G4P23	KT727257				
RVA/Pig-wt/THA/CMP-011-09/2009/G4P6	MG781057				
RVA/Pig/CHN/HuNan-4RV/2021	OR103318				
RVA/Pig/CHN/DB/BL/2306221/2023	PP053640				
RVA/Pig-wt/VNM/14150_53/2012		KX363336			
RVA/Pig/CHN/FX17/2021		OM362099			
RVA/Pig-wt/VNM/12070_4/2012			KX363285		
RVA/Human-wt/VNM/RVN17.0271/2017/G3P23			LC765814		
RVA/Human-wt/ZAF/UFS-NGS-MRC-DPRU2291/2009/G1P8			MT854947		
RVA/Pig/Tanzania/IP058/2019				ON092386	
RVA/Pig/CHN/FJSH01/2021/G26P23				ON093977	
RVA/Pig/CHN/GL/2022				PP003811	
RVA/Human/CHN/R479/2004/G4P6					GU189558
RVA/Pig-tc/CHN/SCMY-A3/2017/G9P23					MK026444

RVA/Human/CHN/LL3354/2000/G5P6				KC139789
RVA/Pig-wt/CHN/CN1P7/2021/G1P7				OP886872
RVA/Pig/CHN/RotaAS143/2019	OM201226			
RVA/pig/CHN/SCGY-192/2018/G5P[13]		MT198767	MT198726	
RVA/Pig/TWN/106-P-002-1-0095/2017/G4P13			MK227894	
RVA/Pig /TWN/105-P-002-1-1237/2016/G4P23			MK227889	
