# Supplementary material for: Isolation, Genomic Characterization and Evolution of Six Porcine Rotavirus A Strains in a Pig Farming Group
Source: Vet Sci. 2024 Sep 14;11(9):436. doi: 10.3390/vetsci11090436 (PMC11435977; doi:10.3390/vetsci11090436)
Supplement: Supplementary file 1 [file vetsci-11-00436-s001.zip › vetsci-3197019-supplementary/supplementary files/Supplementary Tables S3--S13.pdf]

Supplementary Table S3. The nucleotide identity of VP7 gene between the six isolates

VP7	Pairwise % identity					
	AHBZ2303	AHBZ2305	AHBZ2304	AHBZ2312	AHBZ2310	AHBZ2402
AHBZ2303	/	/	/	/	/	/
AHBZ2305	75.4	/	/	/	/	/
AHBZ2304	75.0	98.3	/	/	/	/
AHBZ2312	73.6	75.1	76.6	/	/	/
AHBZ2310	75.2	99.8	98.5	75.1	/	/
AHBZ2402	83.0	77.8	77.6	75.7	77.8	/

Supplementary Table S4. The nucleotide identity of VP4 gene between the six isolates

VP4	Pairwise % identity					
	AHBZ2303	AHBZ2305	AHBZ2304	AHBZ2312	AHBZ2310	AHBZ2402
AHBZ2303	/	/	/	/	/	/
AHBZ2305	73.9	/	/	/	/	/
AHBZ2304	74.1	99.8	/	/	/	/
AHBZ2312	68.1	72.4	66.3	/	/	/
AHBZ2310	73.9	99.7	99.8	72.4	/	/
AHBZ2402	73.4	92.4	92.6	72.3	92.4	/

Supplementary Table S5. The nucleotide identity of VP6 gene between the six isolates

VP6	Pairwise % identity					
	AHBZ2303	AHBZ2305	AHBZ2304	AHBZ2312	AHBZ2310	AHBZ2402
AHBZ2303	/	/	/	/	/	/
AHBZ2305	90.5	/	/	/	/	/
AHBZ2304	89.1	96.0	/	/	/	/
AHBZ2312	82.9	82.9	82.6	/	/	/
AHBZ2310	88.7	95.6	99.3	82.2	/	/
AHBZ2402	89.5	88.9	88.8	82.7	88.1	/

Supplementary Table S6. The nucleotide identity of VP1 gene between the six isolates

VP1	Pairwise % identity					
	AHBZ2303	AHBZ2305	AHBZ2304	AHBZ2312	AHBZ2310	AHBZ2402
AHBZ2303	/	/	/	/	/	/
AHBZ2305	96.6	/	/	/	/	/
AHBZ2304	99.9	96.6	/	/	/	/
AHBZ2312	93.4	92.7	93.3	/	/	/
AHBZ2310	99.9	96.6	100	93.3	/	/
AHBZ2402	86.5	85.6	86.5	86.2	86.5	/

Supplementary Table S7. The nucleotide identity of VP2 gene between the six isolates

VP2	Pairwise % identity					
	AHBZ2303	AHBZ2305	AHBZ2304	AHBZ2312	AHBZ2310	AHBZ2402
AHBZ2303	/	/	/	/	/	/

AHBZ2305	99.3	/	/	/	/	/
AHBZ2304	99.9	99.3	/	/	/	/
AHBZ2312	87.2	86.7	87.2	/	/	/
AHBZ2310	100	99.2	99.9	87.2	/	/
AHBZ2402	96.9	96.3	96.9	87.6	96.9	/

Supplementary Table S8. The nucleotide identity of VP3 gene between the six isolates

VP3	Pairwise % identity					
	AHBZ2303	AHBZ2305	AHBZ2304	AHBZ2312	AHBZ2310	AHBZ2402
AHBZ2303	/	/	/	/	/	/
AHBZ2305	84.8	/	/	/	/	/
AHBZ2304	84.9	99.7	/	/	/	/
AHBZ2312	84.9	99.8	99.8	/	/	/
AHBZ2310	84.9	99.7	99.8	99.8	/	/
AHBZ2402	84.8	86.5	86.6	86.6	86.5	/

Supplementary Table S9. The nucleotide identity of NSP1 gene between the six isolates

NSP1	Pairwise % identity					
	AHBZ2303	AHBZ2305	AHBZ2304	AHBZ2312	AHBZ2310	AHBZ2402
AHBZ2303	/	/	/	/	/	/
AHBZ2305	99.9	/	/	/	/	/
AHBZ2304	99.9	99.9	/	/	/	/
AHBZ2312	98.8	98.7	98.6	/	/	/
AHBZ2310	99.9	99.7	99.7	98.6	/	/
AHBZ2402	82.0	81.9	82.0	81.3	82.0	/

Supplementary Table S10.
The nucleotide identity of NSP2 gene between the six isolates

NSP2	Pairwise % identity					
	AHBZ2303	AHBZ2305	AHBZ2304	AHBZ2312	AHBZ2310	AHBZ2402
AHBZ2303	/	/	/	/	/	/
AHBZ2305	99.9	/	/	/	/	/
AHBZ2304	100	99.9	/	/	/	/
AHBZ2312	89.6	89.5	89.6	/	/	/
AHBZ2310	100	99.9	100	89.6	/	/
AHBZ2402	93.7	93.6	93.7	90.4	93.7	/

Supplementary Table S11.
The nucleotide identity of NSP3 gene between the six isolates

NSP3	Pairwise % identity					
	AHBZ2303	AHBZ2305	AHBZ2304	AHBZ2312	AHBZ2310	AHBZ2402
AHBZ2303	/	/	/	/	/	/
AHBZ2305	99.6	/	/	/	/	/

AHBZ2304	99.9	99.7	/	/	/	/
AHBZ2312	99.7	99.5	99.8	/	/	/
AHBZ2310	99.9	99.7	100	99.8	/	/
AHBZ2402	96.0	95.8	96.1	95.9	96.1	/

Supplementary Table S12.

The nucleotide identity of NSP4 gene between the six isolates

NSP4	Pairwise % identity					
	AHBZ2303	AHBZ2305	AHBZ2304	AHBZ2312	AHBZ2310	AHBZ2402
AHBZ2303	/	/	/	/	/	/
AHBZ2305	98.1	/	/	/	/	/
AHBZ2304	98.1	100	/	/	/	/
AHBZ2312	92.2	91.1	91.1	/	/	/
AHBZ2310	98.1	100	100	99.8	/	/
AHBZ2402	90.7	89.2	96.1	95.9	89.2	/

Supplementary Table S13.

The nucleotide identity of NSP5 gene between the six isolates

NSP5	Pairwise % identity					
	AHBZ2303	AHBZ2305	AHBZ2304	AHBZ2312	AHBZ2310	AHBZ2402
AHBZ2303	/	/	/	/	/	/
AHBZ2305	100	/	/	/	/	/
AHBZ2304	100	100	/	/	/	/
AHBZ2312	99.7	99.7	99.7	/	/	/
AHBZ2310	100	100	100	99.7	/	/
AHBZ2402	97.1	97.1	97.1	96.8	97.1	/
