# Supplementary figures and images for: Isolation, Genomic Characterization and Evolution of Six Porcine Rotavirus A Strains in a Pig Farming Group
Source: Vet Sci. 2024 Sep 14;11(9):436. doi: 10.3390/vetsci11090436 (PMC11435977; doi:10.3390/vetsci11090436)

## Slide 1
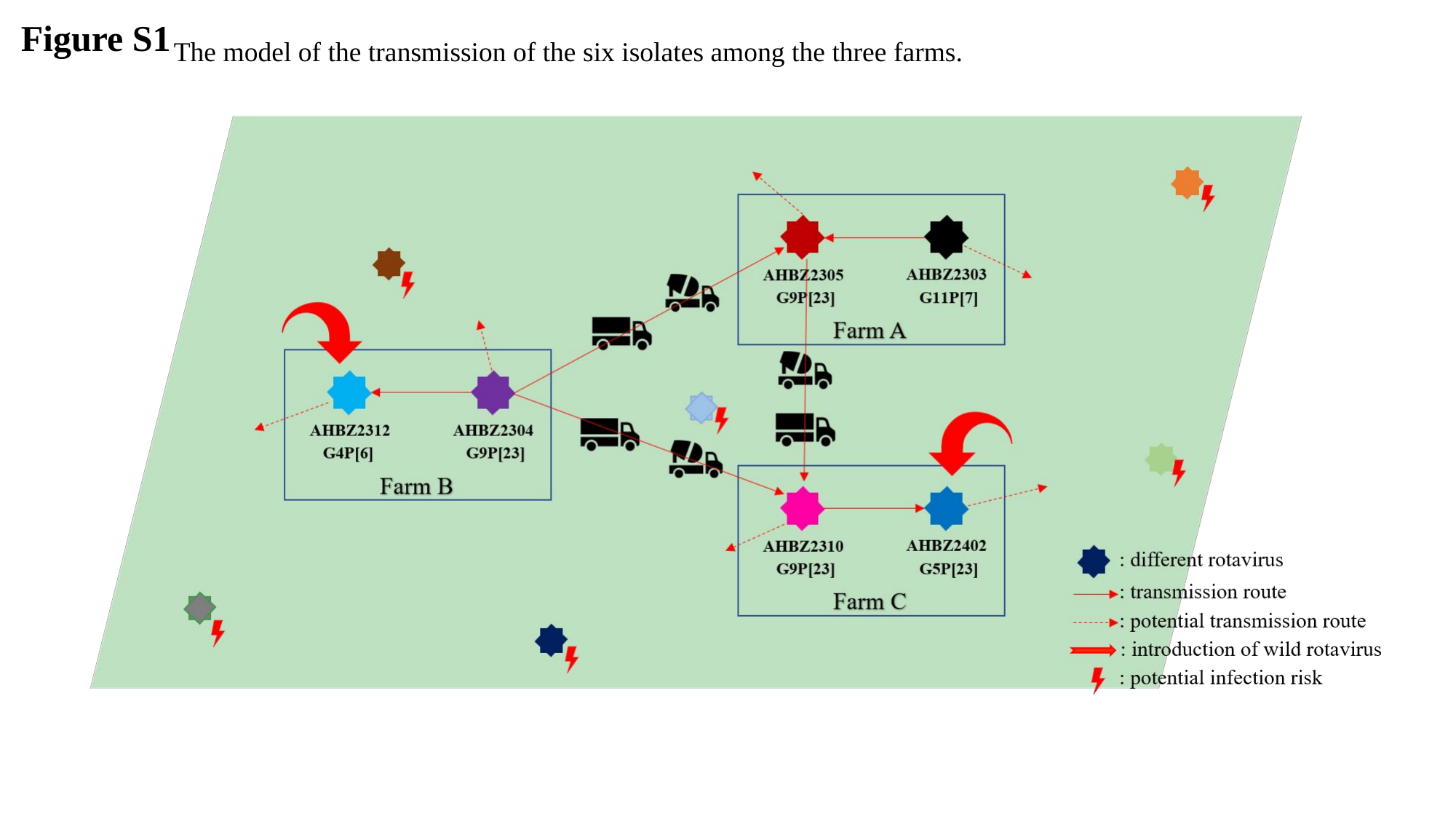

The model of the transmission of the six isolates among the three farms.
Figure S1

Supplement: Supplementary file 1 [file vetsci-11-00436-s001.zip › vetsci-3197019-supplementary/supplementary files/Supplementary Figure S1.pptx]
